# Clinical Characteristics of Patients with Hepatocellular Carcinoma: A Single-Center 3-Year Experience from Somalia

**DOI:** 10.1155/2022/3370992

**Published:** 2022-04-02

**Authors:** Mohamed A. Hassan-Kadle, Marian Muse Osman, Esra Keles, Hasan Huseyin Eker, Kursad Nuri Baydili, Hussein Mahdi Ahmed, Abdirahman Abdikadir Osman

**Affiliations:** ^1^SomGastro Clinic Center for Digestive and Liver Disease, College of Medicine and Health Science, Abrar University, Mogadishu, Somalia; ^2^Department of Public Health, University of Health Sciences Turkey, Mogadishu Somalia-Turkey Recep Tayyip Erdoğan Training and Research Hospital, Mogadishu, Somalia; ^3^Department of Gynecologic Oncology, University of Health Sciences Turkey, Zeynep Kamil Training and Research Hospital, Istanbul 34668, Turkey; ^4^Department of Public Health, University of Health Sciences Turkey, Hamidiye Faculty of Medicine, Istanbul 34668, Turkey; ^5^Department of Biostatistics, University of Health Sciences Turkey, Hamidiye Faculty of Medicine, Istanbul 34668, Turkey; ^6^Department of Internal Medicine, University of Health Sciences Turkey, Mogadishu Somalia-Turkey Recep Tayyip Erdoğan Training and Research Hospital, Mogadishu, Somalia

## Abstract

**Background:**

To evaluate the relationship between prognosticators representing tumor aggressiveness and socio-demographic, laboratory, and imaging findings in patients with hepatocellular carcinoma (HCC).

**Methods:**

We retrospectively searched patients with HCC between January 2017 and December 2019 in our tertiary referral hospital. The tumor-related factors and liver damage indicators and their relationship to indicate the value of prognosis were analyzed.

**Results:**

A total of 268 HCC patients, with a male-to-female ratio of 2.8 : 1. The mean age was 52.6 years. The patient with portal vein thrombosis (PVT) was older, had higher liver laboratory parameters (AST, ALT, total bilirubin, and direct bilirubin), and had larger tumor size. Patients with the larger tumor size had a higher AFP level, had more tumor multifocality. The majority of patients were in Child's A (73.6%) and B (17.2%) classes. The laboratory parameters of HCC patients were increased in Child's C compared to other groups of Child-Pugh classification.

**Conclusions:**

The presence of PVT and large-sized tumor in patients with HCC indicated a poorer prognosis than non-PVT group and small tumor sizes.

## 1. Introduction

Hepatocellular carcinoma (HCC) is the most common primary liver cancer, with more than 1 million deaths expected worldwide in 2030 [1, 2]. The prognostic factors of hepatocellular cancer include demographic characteristics of patients (age, gender, accompanying hepatitis B, C infections, degree of cirrhosis), tumor-related factors (tumor size, tumor multifocality or presence of portal vein thrombosis, AFP values), and liver damage indicators that reflect microenvironment of the liver or tumor such as GGT, ALT, AST, and bilirubin [3–6].

The indicators that reflect the aggressiveness of liver cancer were the presence of portal vein thrombosis (PVT), tumor size, tumor multifocality, and AFP levels. The presence of PVT is an indicator of poor prognosis and is present in 10-40% of HCC patients at the time of diagnosis, and in 35-44% at the time of transplantation or autopsy [7, 8]. Since the presence of PVT constitutes a contraindication for a liver transplant and transarterial chemoembolization, the presence of PVT plays a critical role in HCC treatment. Thus, evaluation of the association between the presence and absence of PVT with clinicopathological features and biochemical variables is crucial in liver cancer treatment.

To date, many studies have been carried out on the effect of tumor size on the selection of treatment regimen, the stage and recurrence of disease, and survival in HCC. Also, tumor size, tumor multifocality, presence of PVT, and Child-Pugh scoring were taken into consideration in prognosticating the survival in the Barcelona Clinic Liver Cancer (BCLC) and other surgical staging systems [9]. Besides a recent population-based study revealed that the utilization of tumor size was beneficial for survival in HCC [10]. Therefore, the size of the tumor, defined as tumor-related prognostic factors, plays an important role in representing the outcome of HCC.

This paper focused on the relationship between prognostic indices including PVT, tumor size, Child-Pugh scoring, and clinical and laboratory parameters in HCC cases. In addition, the aim of our work is to elucidate the relationship between prognosticators representing tumor aggressiveness and laboratory parameters in order to better allocate the treatment management of patients diagnosed with HCC.

## 2. Materials and Methods

Following approval by Ethical Review Committee (13.05.2020-MSTH/3789) hospital electronic medical records were retrospectively searched for patients with hepatocellular cancer diagnosed between January 2017 and December 2019 at the largest tertiary referral hospital in the country. Database management complies with legislation on privacy and this research is in accordance with the ethical principles of the Declaration of Helsinki. Informed consent was waived due to the retrospective study design by the same ethics committee that approved this study.

Abstracted data included tumor size, the number of nodules, tumor sites, the presence of portal vein thrombosis; complete blood counts (white blood cells, hemoglobin, platelets), serum alpha fetoprotein (AFP) value, C-reactive protein (CRP), and albumin; routine blood liver and kidney function tests (alanine aminotransferase [ALT], aspartate transaminase [AST], alkaline phosphatase [ALP], gamma-glutamyltransferase [GGT], total and direct bilirubin, creatinine); blood coagulation system parameters (Standardization of Prothrombin Time/International Normalized Ratio [PT/INR], activated partial thromboplastin time [APTT]), Child-Pugh classification; patients' demographics (age, gender, comorbidities, presence or absence of hepatitis B and C). Information on tumor characteristics was obtained from at least one of the imaging techniques consisting of magnetic resonance imaging (MRI), computed tomography (CT), and sonography. The diagnosis of HCC was made according to the American Association for Liver Disease Research (AASLD) guideline or histopathological examination.

### 2.1. Statistical Analysis

All statistical analyses were made using SPSS (Version 25.0. 2017, IBM SPSS Statistics for Windows; IBM Corp. Armonk, NY, United States of America). Continuous variables with normally distributed data were presented with mean and standard deviation (Mean ± SD), continuous and abnormally distributed data as median (minimum-maximum) where necessary. Categorical variables were expressed as counts and percentages. Chi-square, Mann–Whitney *U* test, and Kruskal-Wallis H test were used to compare groups, where appropriate. Shapiro-Wilk test was used to assess whether the data were normally distributed. In this study, a type I error rate of 0.05 and a *p*-value of less than 0.05 were considered statistically significant.

## 3. Results

A total of 268 hepatocellular cancer (HCC) patients (overall mean age: 52.6 years; *M* : *F* ratio; 2.8 : 1) were recruited during the study period. The HCC patients with PVT were older than those without PVT (55 vs 40 years, *p*-value 0.009). Patients with PVT had higher AST, ALT, total bilirubin, and direct bilirubin levels (*p* = 0.001) compared to HCC patients without PVT. The mean tumor size of patients with PVT was significantly larger than those without PVT (4.0 vs 8.0 cm, *p* = 0.010). Tumor multifocality and the distribution were not significantly different between the two groups. The comparison of HCC patients with and without PVT in respect to demographics, laboratory results, and imaging findings is demonstrated in Table 1.

Patients were divided into three groups according to their maximal tumor diameter (MTD). We observed that the larger size tumors had more likely to have high AFP levels (*p* < 0.001). The multifocality of the tumor was significantly higher in the large size tumor groups than in the small size tumor groups (55% vs 15% vs 28% in the 0.1–4.9 vs 5.1–9.9 vs ≥10.0 groups, respectively, all *p* = 0.006). Increased numbers of metastatic tumor nodules and distribution of tumors were observed in the larger size groups. The distribution of tumor was significantly higher in large size tumor groups compared to small-sized tumor groups (all *p* = 0.007).

There was no significant difference in demographic characteristics, laboratory findings, the presence and absence of cirrhosis, and PVT. The distribution of patients with HCC according to maximal tumor size is shown in Table 2.

The total study group was divided accordingly to the Child-Pugh classification. Among 266 HCC patients, 73.6% (196/266) of patients were Child's A, 17.2% (46/266) were Child's B, and 9.0% (24/266) were Child's C. Concerning the demographic characteristics and clinical data of HCC patients, there were no differences between the groups except for the hepatitis B infection. Regarding the tumor-related parameters of patients diagnosed with HCC, maximal tumor size and presence and absence of PVT were significantly different between the three groups. In addition, laboratory findings including ALT, AST, ALP, GGT, total bilirubin, direct bilirubin, and PT/INR levels were significantly higher in Child-Pugh C compared to other groups (all *p* < 0.001; Table 1). Therefore, as the Child-Pugh score increases, laboratory findings including ALT, AST, ALP, GGT, total bilirubin, direct bilirubin, and PT/INR levels also significantly increase. The comparison of the characteristics of tumor-related parameters, demographic features of patients, and imaging examination findings with the Child-Pugh classification is shown in Table 3.

## 4. Discussion

Liver cancer is the third leading cause of cancer deaths worldwide, with approximately 905 677 (4.7%) of all new cases, leading to 830 180 (8.3%) deaths in 2020, ranks fifth among males and seventh among females. Hepatocellular carcinoma is primary liver cancer, which accounts for 85–90% of liver cancers and is the sixth most common cancer globally [11, 12]. This paper describes for the first time the demographic characteristics, risk factors, laboratory parameters, imaging findings, and prognostic factors of HCC patients from the largest referral hospital in Somalia before and after the civil war. In sub-Saharan Africa, the Asia-Pacific region showed male dominance over females by a ratio of about 3 : 1 to 4 : 1 [13]. In this study, HCC patients were much higher in males compared to females with a ratio of 2.8 : 1. The predominance of males lent support to previous findings [14]. Our results are in line with studies reported from countries in the region, such as Ghana [15] and Egypt [16], where male domination was reported. The male predominance demonstrated in these studies was explained by the higher susceptibility of males to environmental carcinogens and greater exposure to environmental risk factors compared to the female population [16]. Our study showed that the mean age of patients diagnosed with HCC was 52.6 years fits well with the epidemiological review by MC [13] on sub-Saharan Africa. The values were also consistent with earlier studies in Ghana [15] and Egypt [16].

There are factors that are prognostically important in HCC which are categorized into groups as hepatocellular-related factors (liver function tests, bilirubin) and tumor aggressiveness related factors (tumor size, presence of PVT, elevated AFP levels, and tumor multifocality). The current study has focused on these prognosticators. HCC commonly invades into the portal vein in most patients with advanced-stage HCC. PVT was diagnosed by computed tomography, magnetic resonance imaging, or sonography, showing portal vein obstruction and dilation. PVT is a common complication of HCC that has been associated with a poor prognosis [17]. Our study demonstrated that 15.2% (41/268) of HCC patients had portal vein thrombosis who were older and higher liver function values (AST, ALT, bilirubin) than those without PVT. These values correlate well with Carr et al. [18] and further support the concept of higher bilirubin due to more aggressive tumors causing parenchymal destruction or due to increased PVT in patients.

In the presented study, PVT positivity was found to be associated with larger tumor size, consistent with studies showing that the presence of PVT is related to a larger tumor size than those without PVT. There are several possible explanations for the presence of PVT in larger tumor sizes. It can thus be conceivably hypothesized the stimulation of tumor growth factors such as stem cells or growth factors. Another possible explanation may be the increasing invention of the tumor factor and its causes of portal vein tumor thrombosis which is a poor prognostic factor [19].

The tumor-related factors are one main prognostic factor of HCC patients such as tumor size. The association between tumor size and AFP level is noteworthy because we found that the larger the tumor size, the higher the level of AFP. This is in good agreement with a recent study by Akkiz et al. [20], which indicated higher levels of AFP in larger tumors rather than small tumor sizes.

One of the more significant findings to emerge from this study is that the distribution of Child-Pugh classification was 73.6% (196/266) of patients were Child's A, 17.2% (46/266) were Child's B, and 9.0% (24/266) were Child's C. A recent ten-year study by Mekonnen et al. [21] reported that Child-Pugh scoring system distribution was 12 (44.4%) Child's A, 12 (44%) Child's B, and only 3 (11.2%) of Child's C. The comprehensive study of Abd-Elsalam et al. [16], including 1440 HCC patients, revealed that the distribution of 37.8% of patients was Child's A, 35.3% were Child's B, and 26.9% were Child's C. The present study confirms previous findings and contributes to the growing body of research that suggests Child-Pugh classification is important in furthering our understanding of the role of the assessment of the severity of liver dysfunction. The conspicuous observation to emerge from the data comparison was the correlation between liver function tests, coagulation parameters, and the Child-Pugh classification system because these laboratory parameters increase as the Child-Pugh classification progresses. Our results share a number of similarities with Siddiq et al.'s findings [22].

We are aware that our research may have several limitations. The most important limitation lies in the fact that it is the single-center experience. Although the current study is conducted in a single center, it is the largest tertiary healthcare facility offering comprehensive and referral-level care in the country. The second is the clinicians' lack of awareness of the disease; the burden of healthcare costs on the patient makes it extremely difficult to obtain complete data. Another limitation is the difficulties experienced in the pathological confirmation process of this cancer due to the lack of surgical equipment required for biopsy and the lack of experienced healthcare personnel. Despite these limitations, a key strength of the current study is that it represents the first comprehensive study investigating hepatocellular cancer before and after the Somalia civil war.

## 5. Conclusion

This is the first large-scale study on patients with HCC cancer in our setup before and after the civil war which is related to prognostic factors of HCC. The findings of this study have several important implications for clinical practice in low-source settings guiding both clinicians and healthcare providers to develop a deeper understanding of liver cancer. We have obtained comprehensive results proving the presence of PVT and large-sized tumors indicating a poorer prognosis compared to the non-PVT group and small tumor sizes. Somalis patients mostly attending to the hospitals at the advanced stage of disease result in a poor prognosis. One of the etiologic causes of hepatocellular cancer is hepatitis B infection which the prevalence of hepatitis B infection in Somalia is 18% [23]. Therefore, our findings suggest several courses of action for diagnosing HCC cancer in the early stages. A reasonable approach to tackle this preventable disease could be to expanding hepatitis B vaccination, enhancing the awareness of the hepatitis infection, and increasing health literacy. Moreover, this broad research finding also points to the need for a cancer care unit in our country to get development programs for cancer control and prevention. Thus, it is necessary to initiate a national cancer care unit. A key national policy priority should therefore be to prevent viral hepatitis infections in order to prevent the HCC and its consequences. Continued efforts are needed to make vaccination more accessible in the country.

## Figures and Tables

**Table 1:** Comparison of hepatocellular carcinoma (HCC) patients by portal vein thrombosis (PVT+/-) (*n* = 268), Jan 2017–Dec 2019, Somalia.

	PVT (-) (*n* = 227)	PVT (+) (*n* = 41)	*p*
Gender *N* (%)			
*Male*	163 (82.7)	34 (17.3)	0.210
*Female*	63 (90)	7 (10)	
Age (median)(years)	40 (22-76)	55 (18-100)	**0.009**
AFP	2000 (201-2000)	2000 (203-2000)	**0.014**
AST (U/L)	97 (0-4483)	216 (0-16335)	**0.001**∗
ALT (U/L)	39 (0-1269)	77 (0-3456)	**0.001**∗
Creatinine (mg/dl)	0.6 (0-5.1)	0.7 (0-8.4)	0.206
Direct_Bilirubin (*μ*mol/L)	0.2 (0-93)	1.2 (0-28.43)	**0.001**∗
Protrombin time (seconds)	0 (0-61.9)	0 (0-59.5)	0.230
Total_Bilirubin (*μ*mol/L)	0.55 (0-30)	3.3 (0-34.87)	**<0.001**∗
Albumin (g/L)	2.85 (0-5.1)	2.7 (0-4.4)	0.836
GGT (U/L)	0 (0-987)	36 (0-876)	0.202
ALP (U/L)	0 (0-1325)	178 (0-1750)	**0.023**∗
Hb (g/dl)	7 (0-15)	8 (0-13)	0.637
Platelet (×109/L)	70 (0-996)	72.5 (0-876)	0.657
WBC×103	9 (0-987)	13 (0-86)	0.270
CRP	54 (0-987)	65 (0-865)	0.355
Tumor size (cm)	4 (0-27)	8 (0-30)	**0.010**∗
Child-Pugh			
*A*	175 (89.3)	21 (10.7)	**0.001**∗
*B*	33 (71.7)	13 (28.3)	
*C*	17 (70.8)	7 (29.2)	
Cirrhosis (-) (%)	163 (86.7)	25 (13.3)	0.195
Cirrhosis (+) (%)	62 (79.5)	16 (20.5)	
Number of nodules			
*Unifocal*	34 (79.1)	9 (20.9)	0.703
*Multifocal*	33 (73.3)	12 (26.7)	
Tumor distribution			
*Unilobular*	199 (85.4)	34 (14.6)	0.515
*Bilobular*	27 (79.4)	7 (20.6)	
Maximal tumor size (cm)			
*<5* cm	111 (89.5)	13 (10.5)	0.087
*5-10*	32 (84.2)	6 (15.8)	
*>10*	83 (79)	22 (21)	

Abbreviations: ALT: alanine aminotransferase; AST: aspartate aminotransferase; PT: prothrombin time; GGT: gamma glutamyl transpeptidase; INR: international normalized ratio; PT: prothrombin time; AFP: alpha fetoprotein; WBCs: white blood cells; CRP: C-reactive protein; normal values: ALT (5–45 U/L), AST (5–40 U/L), alkaline phosphatase (38–155 U/L), albumin (2.5–6.0 g/dL), total serum bilirubin (0.1–1.3 mg/dL), and serum direct bilirubin (0.0–8.4 mg/dL).

**Table 2:** Comparison of hepatocellular carcinoma (HCC) patients by maximal tumor diameter categories (*n* = 267), Jan 2017–Dec 2019, Somalia.

	MTD			
	<5 cm (*n* = 124)	5-10 cm (*n* = 38)	≥10 cm (*n* = 105)	*p*
Gender, *N* (%)				
*Male*	86 (43.7)	28 (14.2)	83 (42.1)	0.242
*Female*	38 (54.3)	10 (14.3)	22 (31.4)	
Age (median)(years)	52 (18-100)	55 (20-91)	53 (20-90)	0.696
AFP	1046.14 (201-2000)	2000 (203-2000)	2000 (223-2000)	**<0.001**∗
AST (U/L)	95 (0-16335)	97 (0-4483)	120 (0-3432)	0.175
ALT (U/L)	40.5 (0-3456)	41.5 (0-1260)	42 (0-477)	0.750
Creatinine (mg/dl)	0.6 (0-8.4)	0.8 (0-2.5)	0.7 (0-7.8)	0.334
Direct bilirubin (*μ*mol/L)	0.25 (0-93)	0.1 (0-17.7)	0.3 (0-22.7)	0.784
PT (seconds)	0 (0-59.5)	0 (0-48.6)	0 (0-61.9)	0.652
Total bilirubin (*μ*mol/L)	0.6 (0-34.87)	0.45 (0-22.94)	0.9 (0-29.4)	0.333
Albumin (g/dL)	2.7 (0-4.7)	3.15 (0-5)	2.8 (0-5.1)	0.259
GGT (U/L)	0 (0-987)	0 (0-321)	17 (0-768)	0.290
ALP (U/L)	0 (0-1750)	0 (0-743)	113 (0-1157)	0.186
Hb (g/dl)	8 (0-15)	7 (0-14)	7 (0-13)	0.575
Platelet (×109/L)	67 (0-996)	85 (0-980)	67 (0-876)	0.204
WBC×103	12 (0-987)	8 (0-97)	9 (0-90)	0.709
CRP	58 (0-876)	65 (0-987)	56 (0-879)	0.741
Child-Pugh				
*A*	90 (45.9)	28 (14.3)	78 (39.8)	**0.014**∗
*B*	16 (34.8)	10 (21.7)	20 (43.5)	
*C*	18 (75)	0 (0)	6 (25)	
Cirrhosis (-) (%)	94 (50)	23 (12.2)	71 (37.8)	0.153
Cirrhosis (+) (%)	30 (38.5)	15 (19.2)	33 (42.3)	
PVT (-)	111 (49.1)	32 (14.2)	83 (36.7)	0.091
PVT (+)	13 (31.7)	6 (14.6)	22 (53.7)	
Number of nodules				
*Unifocal*	27 (62.8)	5 (11.6)	11 (25.6)	**0.006**∗
*Multifocal*	13 (28.9)	7 (15.6)	25 (55.6)	
Tumor distribution				
*Unilobular*	116 (49.8)	29 (12.4)	88 (37.8)	**0.007**∗
*Bilobular*	8 (23.5)	9 (26.5)	17 (50)	

Abbreviations: ALT: alanine aminotransferase; AST: aspartate aminotransferase; PT: prothrombin time; GGT: gamma glutamyl transpeptidase; INR: international normalized ratio; :PT: prothrombin time; AFP: alpha fetoprotein; WBCs: white blood cells; CRP: C-reactive protein; PVT: portal vein thrombosis; normal values: ALT (5–45 U/L), AST (5–40 U/L), alkaline phosphatase (38–155 U/L), albumin (2.5–6.0 g/dL), total serum bilirubin (0.1–1.3 mg/dL), and serum direct bilirubin (0.0–8.4 mg/dL).

**Table 3:** Characteristics of hepatocellular carcinoma (HCC) patients by Child-Pugh Score (*n* = 266), Jan 2017–Dec 2019, Somalia.

	Child A (*n*, %)	Child B (*n*, %)	Child C (*n*, %)	*p*
Age	55 (18-100)	49 (20-87)	38 (22-88)	0.050
Gender				
*Male*	143 (73)	34 (17.3)	19 (9.7)	0.863
*Female*	53 (75.7)	12 (17.1)	5 (7.1)	
Etiology				
*HBV*	66 (60.6)	28 (25.7)	15 (13.8)	**<0.001**∗
*HCV*	31 (75.6)	8 (19.5)	2 (4.9)	0.634
*Non-HBV non-HCV*	47 (17.6)	44 (16.7)	24 (9.1)	0.091
*HBV + HCV co-infection*	1 (33.3)	2 (66.7)	0 (0)	0.091
Comorbidities				
*Diabetes mellitus*	10 (83.3)	2 (16.7)	0 (0)	0.782
*Malaria*	19 (61.3)	6 (19.4)	6 (19.4)	0.459
*Tuberculosis*	19 (61.3)	6 (19.4)	6 (19.4)	0.482
*HIV*	9 (60)	3 (20)	3 (20)	0.185
*Cirrhosis*	54 (69.2)	14 (17.9)	10 (12.8)	0.352
Imaging findings				
Number of nodules				
*Unifocal*	29 (67.4)	10 (23.3)	4 (9.3)	0.384
*Multifocal*	32 (71.1)	6 (13.3)	7 (15.6)	
Maximal tumor size (cm)				
*<5* cm	90 (72.6)	16 (12.9)	18 (14.5)	**0.013**∗
*5-10*	28 (73.7)	10 (26.3)	0 (0)	
*>10*	78 (75)	20 (19.2)	6 (5.8)	
Tumor distribution				
*Unilobular*	169 (72.5)	43 (18.5)	21 (9)	0.422
*Bilobular*	27 (81.8)	3 (9.1)	3 (9.1)	
PVT (-)	175 (77.8)	33 (14.7)	17 (7.6)	**0.002**∗
PVT (+)	21 (51.2)	13 (31.7)	7 (17.1)	
Laboratory results				
Hemoglobin g/dl	7 (0-15)	7 (3-14)	6.5 (4-12)	0.785
Platelets ×109	73 (0-996)	75.5 (3-876)	65 (6-578)	0.795
WBC ×103	9 (0-987)	14 (2-98)	9 (5-856)	0.534
ALT (U/L)	35 (0-3456)	64.5 (0-1260)	227 (28-1476)	**<0.001**∗
AST (U/L)	81.5 (0-16335)	183.5 (0-4483)	451 (143-10068)	**<0.001**∗
ALP (U/L)	0 (0-1003)	123 (0-805)	455.5 (0-1750)	**<0.001**∗
GGT (U/L)	0 (0-987)	27.5 (0-768)	64 (0-765)	**0.001**∗
Albumin (g/dl)	3 (0-5.1)	2.7 (0-4.4)	2.5 (0-4)	0.721
Creatinine (mg/dl)	0.6 (0-5.1)	0.7 (0-8.3)	0.8 (0.2-8.4)	**0.006**∗
AFP	2000 (201-2000)	2000 (231-2000)	1147.92 (205-2000)	0.199
CRP	53 (0-876)	66.5 (0-987)	75 (5-798)	0.050

Abbreviations: ALT: alanine aminotransferase; AST: aspartate aminotransferase; PT: prothrombin time; GGT: gamma glutamyl transpeptidase; INR: international normalized ratio; :PT: prothrombin time; AFP: alpha fetoprotein; WBCs: white blood cells; CRP: C-reactive protein; PVT: portal vein thrombosis; normal values: ALT (5–45 U/L), AST (5–40 U/L), alkaline phosphatase (38–155 U/L), albumin (2.5–6.0 g/dL), total serum bilirubin (0.1–1.3 mg/dL), and serum direct bilirubin (0.0–8.4 mg/dL).

## Data Availability

The data used to support the findings of this study are available from the corresponding author upon request.

## Additional Points

*Main Points*. (i) The patient with PVT was older, had elevated liver parameters, larger tumor, and more tumor foci. (ii) The presence of PVT and large-sized tumor indicated a worse prognosis than non-PVT group. (iii) This is the first large-scale study investigating hepatocellular carcinoma before and after the Somalia civil war.
